# Towards functional robotic training: motor learning of dynamic tasks is enhanced by haptic rendering but hampered by arm weight support

**DOI:** 10.1186/s12984-022-00993-w

**Published:** 2022-02-13

**Authors:** Özhan Özen, Karin A. Buetler, Laura Marchal-Crespo

**Affiliations:** 1 Motor Learning and Neurorehabilitation Laboratory, ARTORG Center for Biomedical Engineering Research, University of Bern, Freiburgstrasse 3, 3010 Bern, Switzerland; 2 Department of Cognitive Robotics, Delft University of Technology, Mekelweg 2, 2628 CD Delft, the Netherlands

**Keywords:** Motor learning, Motor control, Neurorehabilitation, Somatosensory information, Haptic rendering, Robotic assistance, Arm weight support, Variability, Effort, Motivation

## Abstract

**Background:**

Current robot-aided training allows for high-intensity training but might hamper the transfer of learned skills to real daily tasks. Many of these tasks, e.g., carrying a cup of coffee, require manipulating objects with complex dynamics. Thus, the absence of somatosensory information regarding the interaction with virtual objects during robot-aided training might be limiting the potential benefits of robotic training on motor (re)learning. We hypothesize that providing somatosensory information through the haptic rendering of virtual environments might enhance motor learning and skill transfer. Furthermore, the inclusion of haptic rendering might increase the task realism, enhancing participants’ agency and motivation. Providing arm weight support during training might also enhance learning by limiting participants’ fatigue.

**Methods:**

We conducted a study with 40 healthy participants to evaluate how haptic rendering and arm weight support affect motor learning and skill transfer of a dynamic task. The task consisted of inverting a virtual pendulum whose dynamics were haptically rendered on an exoskeleton robot designed for upper limb neurorehabilitation. Participants trained with or without haptic rendering and with or without weight support. Participants’ task performance, movement strategy, effort, motivation, and agency were evaluated during baseline, short- and long-term retention. We also evaluated if the skills acquired during training transferred to a similar task with a shorter pendulum.

**Results:**

We found that haptic rendering significantly increases participants’ movement variability during training and the ability to synchronize their movements with the pendulum, which is correlated with better performance. Weight support also enhances participants’ movement variability during training and reduces participants’ physical effort. Importantly, we found that training with haptic rendering enhances motor learning and skill transfer, while training with weight support hampers learning compared to training without weight support. We did not observe any significant differences between training modalities regarding agency and motivation during training and retention tests.

**Conclusion:**

Haptic rendering is a promising tool to boost robot-aided motor learning and skill transfer to tasks with similar dynamics. However, further work is needed to find how to simultaneously provide robotic assistance and haptic rendering without hampering motor learning, especially in brain-injured patients.

*Trial registration* https://clinicaltrials.gov/show/NCT04759976

**Supplementary Information:**

The online version contains supplementary material available at 10.1186/s12984-022-00993-w.

## Background

Every year, millions of stroke survivors lose their functional autonomy due to arm paralysis [1]. In the absence of a cure for stroke, clinical evidence suggests that patients should engage in functional task-specific [2], high-intensity [3] training to maximize their recovery. Rehabilitation robots can provide high-intensity training [4], but current robots seem to hamper the regain of functional movements needed to perform Activities of Daily Living (ADL) [5, 6]. A possible rationale behind this limitation is that current robot-aided interventions rely on (rather abstract) visual feedback while the somatosensory feedback from the interaction with tangible virtual objects is neglected [7].

Training with robots that do not incorporate somatosensory feedback does not resemble real-life training conditions. As we do not expect people to learn how to ride a bike on a static bike or learn how to swim outside the water, how can we expect brain-injured patients to relearn functional movements if we do not allow them to see and feel the interaction with realistic, highly dynamic virtual environments? Yet, a considerable amount of ADLs, such as carrying a cup of coffee, require the physical interaction with objects that have complex dynamics [8]—e.g., non-linear, under-actuated, and even unstable dynamics. During this interaction, humans apply forces on the objects to manipulate them, and objects apply forces back to the humans according to their specific dynamics. The somatosensory information regarding these interaction forces, which is perceived through proprioceptive and tactile mechanoreceptors, plays an essential role in fine motor control [9, 10]. Furthermore, fMRI studies have highlighted the importance of somatic information for forming internal models of object dynamics—e.g., strong activation was observed in brain areas associated with learning, namely the cerebellum, when interacting with objects with complex dynamics [11, 12]. Importantly, when the somatosensory cortex is disrupted, learning of a new task is impaired [13] and the recovery after stroke is hampered [14]. These findings indicate that somatosensory information is important not only for motor control but also for motor (re)learning [15]. Thus, providing somatosensory information (regarding the physical interaction with virtual objects) during robotic therapy might enhance motor learning and neurorehabilitation outcomes [16].

Somatic feedback could be potentially provided by robots through haptic rendering—i.e., by simulating the interaction forces from tangible virtual objects to the participants according to their dynamic models. The provision of congruent visuo-haptic feedback might provide a more realistic training environment that might enhance the skill transfer gained during robotic rehabilitation to ADLs [7, 17]. Importantly, the provision of multimodal feedback has been shown to enhance motor learning on highly realistic virtual reality-based complex tasks [18, 19]. A more realistic and naturalistic virtual environment might also enhance the sense of agency—i.e., the feeling of control over own actions—and patients’ motivation, which are considered to be associated with enhanced motor learning [20–22]. Furthermore, haptic rendering has been shown to increase participants’ workspace exploration [23], crucial to enhance motor learning [24–26].

However, most current robotic therapies neglect the provision of somatosensory feedback during movement training by not providing the haptic rendering of virtual training environments [27, 28]. One reason is that while current heavily constructed exoskeletons enable the training of multijoint movements, their low transparency limits their capability to provide somatosensory stimulation through haptic rendering. Furthermore, by using robotic assistance methods to support patients’ movements [4, 29], the patients’ perception of the haptic rendering might be hampered [30].

Nevertheless, robotic assistance is necessary to support brain-injured patients who cannot generate sufficient force to move their limbs. Furthermore, robotic assistance might benefit motor learning by reducing the tasks’ challenge level during practice [31], enhancing patients’ motivation [32]. Moreover, robotic assistance might limit patients’ fatigue during training [33], allowing higher-intensity therapy [4]. To minimize the potential interference between the haptic rendering and assisting forces, robotic assistance methods that do not affect general patterns of muscle activation (while reducing the average activation level) such as (human) arm weight support [34, 35] might be a better solution compared to movement-constraining methods such as haptic guidance [24]. Arm weight support methods limit muscle fatigue [36], increase patients’ movements workspace [37], and enhance their ability to generate quick coordinated motions [38], which is essential for successfully performing dynamic tasks. Furthermore, limiting the effort that patients need to put into counterbalancing gravity might allow them to direct their efforts towards more effective interactions with the dynamic environment [23].

**Fig. 1:**
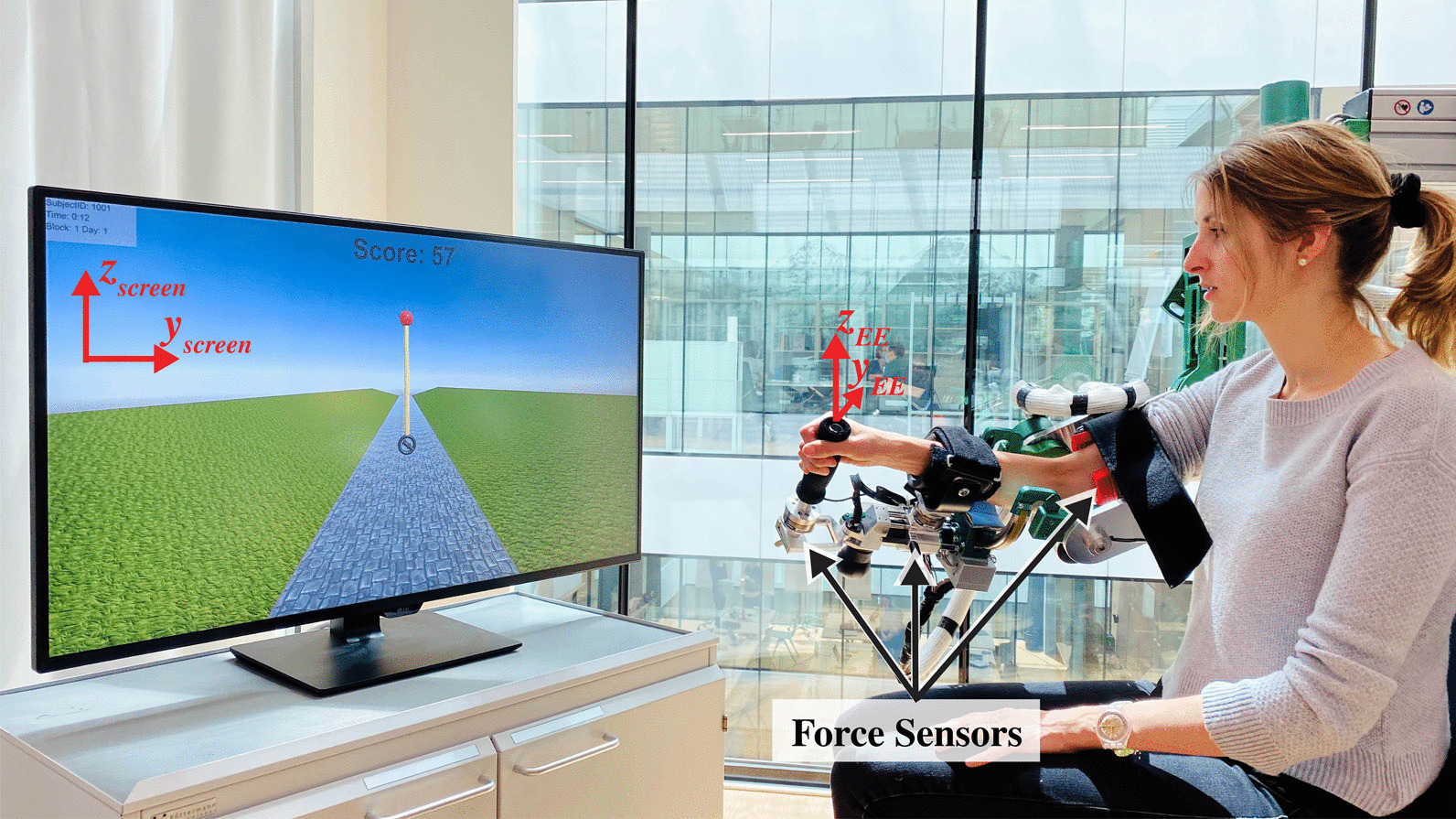
Experimental setup with the ARMin exoskeleton robot. The motor task consisted of inverting a pendulum and keeping it inverted. The pivot point of the pendulum was matched to the movement of the hand module of the robot. The force/torque sensors below the lower/upper arm cuffs and below the hand module allowed participants to move the robot transparently through the use of disturbance observers

To evaluate the effects of haptic rendering in robot-aided motor learning of a complex dynamic task, we conducted a between-subjects factorial design study with 40 healthy participants. We analyzed the effects of haptic rendering, arm weight support, and the interaction of these two factors on motor learning. The highly dynamic motor task consisted of inverting a virtual pendulum by moving their pivot point with the end-effector of a six-degrees-of-freedom (DoF) arm exoskeleton rehabilitation robot (ARMin; Fig. 1). During training, participants either felt the haptic rendering forces of the pendulum dynamics on the hand module or not, and were either provided arm weight support or not, depending on the group they were assigned to. We evaluated learning in the environment closest to reality, namely, in the presence of haptic rendering but without robotic assistance. Importantly, skill transfer was evaluated in a similar task: inverting a pendulum with different dynamics. We assessed the participants’ ability to invert the pendulum, their ability to synchronize their hands’ movements with the pendulum mass, participants’ movement variability, and their physical effort. Furthermore, participants’ sense of agency and motivation were assessed with questionnaires.

We hypothesized that:

1. Haptic rendering would encourage participants’ workspace exploration—i.e., higher movement variability—and physical effort during training compared to training without haptics.
2. Arm weight support would reduce participants’ physical effort and promote workspace exploration during training, compared to non-assistance.
3. There would not be any interaction effects of haptic rendering and arm weight support on the participants’ movement.
4. Training with haptic rendering would enhance motor learning, compared to training without haptics. This enhanced learning would be associated with a change in the participants’ ability to synchronize their movements with the pendulum dynamics.
5. Training with haptic rendering would enhance skill transfer due to the congruent visuo-haptic information during training.
6. Training with arm weight support would enhance learning, compared to training without, due to a more efficient allocation of participants’ effort into the dynamic environment and limited fatigue.
7. Finally, the inclusion of haptic rendering would increase the task realism, enhancing participants’ level of agency and motivation.

## Methods

### Experimental setup

The six DoF arm exoskeleton rehabilitation robot ARMin was employed for this experiment (Fig. 1) [39]. The actuated DoFs are: shoulder elevation, abduction/adduction, internal/external rotation, elbow flexion, forearm supination/pronation, and wrist extension/flexion. To measure the interaction forces between the human and robot, three force/torque sensors (Mini45, ATI Industrial Automation, USA) were located at the hand module and the upper and lower arm cuffs, where the participant’s arm is attached to the exoskeleton (Fig. 1). The motion control of ARMin and simulation of pendulum dynamics were performed with Simulink Realtime R2017b (MathWorks, Massachusetts, USA) at 3 kHz.

The Unity3D game engine (Unity Technologies, USA) was employed to implement the virtual environment (VE) and the experimental protocol. The VE was displayed on an LED screen (109 cm, 43UD79, LG, South Korea) located in front of the participant. The robot motion control and the game software communicated with UDP protocol.

### Pendulum dynamics

**Fig. 2:**
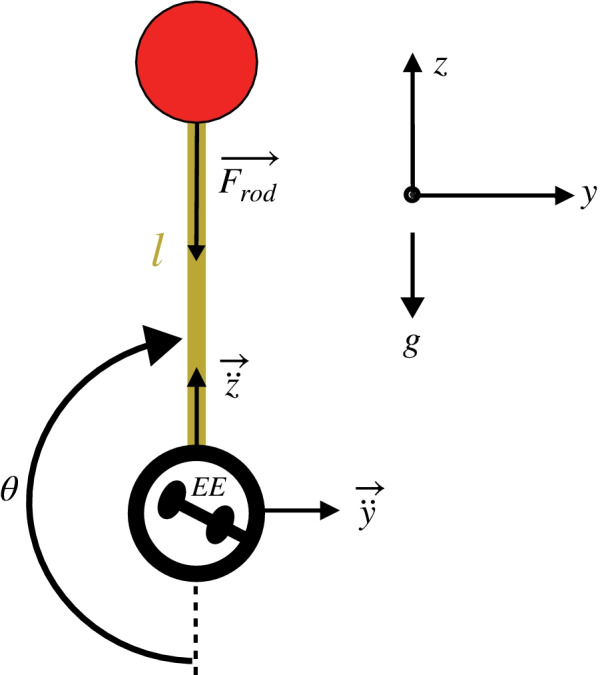
Pendulum dynamics. The *m*, *l*, and *g* represent the pendulum mass, rod length, and gravity, respectively. The pendulum pivot point (EE) was matched to the robot hand module (a stick). The haptic rendering forces from the pendulum dynamics ($F_{rod}$) were applied at the robot hand module. The pendulum is depicted in its unstable equilibrium position

The task to be learned consisted of inverting a virtual pendulum by moving the exoskeleton hand module. The position and orientation of the robot hand module were mapped to the virtual pendulum pivot point with an approximate scaling factor of 0.5. The virtual pendulum pivot point is depicted as a handle inside the black circle of the pendulum in Fig. 2.

Participants could move and rotate the hand module in 3D, but only the movements in the vertical plane affected the pendulum movement. The rotation of the hand module, although did not influence the pendulum movement, still matched with the virtual pendulum handle depicted within the pendulum pivot point (EE in Fig. 2) to facilitate the understanding of the robot-pendulum interface. While only the movement in the horizontal direction would be sufficient to invert the pendulum, we allowed movements in the vertical direction to analyze the effect of the arm weight support on the participants’ movements in the direction of gravity.

The pendulum dynamics had only one internal DoF: the pendulum angle ($\theta$). The pendulum motion was simulated according to the following equation:

1 $$\begin{aligned} \ddot{\theta } = - \frac{1}{l} \bigg ( \ddot{y} \cos \theta + (\ddot{z} + g) \sin \theta \bigg ) - \frac{c}{ml^2}{\dot{\theta }} \end{aligned}$$

where *y* and *z* are the horizontal and vertical directions, respectively. The pendulum mass (*m*) and rod length (*l*) were selected as 2.5 kg and 0.35 m (0.25 m for the transfer task). The damping coefficient (*c*= 0.16 N.s/rad) was required to stabilize the pendulum. We wanted to keep the velocity/acceleration-induced forces of the pendulum (e.g., centrifugal and inertial forces) high, so that the participants could better feel that the pendulum reacts to their actions. To do this, we could either increase the mass—making the pendulum heavier and thus, increasing excessively the participants’ fatigue and limiting the experiment duration—or reduce the gravity so that the pendulum is not too heavy while the mass is high. Therefore, we reduced the gravity coefficient (*g*) to 25% of the real earth gravity.

### Haptic rendering

The haptic rendering forces of the pendulum in the direction of the pendulum rod were calculated according to the following equation:

2 $$\begin{aligned} F_{rod} = m \bigg ({\dot{\theta }}^2 l - \ddot{y} \sin \theta + (g+\ddot{z}) \cos \theta \bigg ). \end{aligned}$$

The $F_{rod}$ was rendered at the robot hand module and transmitted to all robot joints using the hand module Jacobian. An invisible virtual safety table was also haptically rendered at the participants’ leg level to prevent collisions. A warning was presented on the screen every time participants touched the (invisible) table and were prompted to elevate the pendulum.

To reduce the variability in the joint null space and facilitate playing the game, the wrist flexion and shoulder internal rotation were fixed at 0$^\circ$ and 30$^\circ$ with position controllers. The position controllers were soft enough ($\hbox {K}_p$ = 100 N.m.kg$^{-1}$ rad$^{-1}$, $\hbox {K}_d$ = 20 N.m.s.kg$^{-1}$ rad$^{-1}$) to make participants feel the haptic rendering forces of the pendulum in all arm joints.

A Disturbance Observer (DOB) was implemented for each robot joint to compensate for the robot disturbances (e.g., friction, robot weight) and allow the robot to follow the participants’ self-generated movements transparently [39]. The DOBs employed the force/torque measurements as input. However, please note that the perception of the pendulum haptic rendering was still slightly affected by the transparency of the robot as the robot inertia could not be fully compensated.

### Arm weight support

We chose arm weight support as the assisting method because this type of assistance does not depend on the movement, and therefore, we expected minimal interaction between the supporting forces and the haptic rendering. If any interaction between the haptic rendering and the arm weight support was to be observed, this would most probably be more evident when the robot supports completely the weight of the participants’ arms, and therefore, we decided to support 100 $\%$ of the participants’ arm weight.

The arm weight support method—first presented in [40]—uses a model of the participant’s arm to cancel the effect of gravity regardless of the arm pose. The method requires that the individual upper/lower arm weights are estimated. This estimation was performed at the beginning of the experiment, asking each participant to keep for 10 s a fixed pose [−10$^\circ$ shoulder elevation, −10$^\circ$ abduction, 30$^\circ$ external rotation, −30$^\circ$ elbow flexion, 0$^\circ$ forearm supination, and 0$^\circ$ wrist extension]. This fixed pose was selected to maximize the accuracy of the parameter estimation [40].

### The inverted pendulum task

The experimental task consisted of inverting the pendulum and keeping it vertically inverted as long as possible ($\theta = \pi$ rad, ${\dot{\theta }} =$ 0 rad/s) by moving the robot hand module. A score was shown on the screen as feedback to the participants. The score increased according to the following equation:

3 $$\begin{aligned} &\text {Score} = \int _{inv_{begin}}^{inv_{end}} k (\pi /6 -|\theta -\pi |) dt, \text { if:}\\&|\theta -\pi |< \pi /6 \text { rad, and } |{\dot{\theta }}| < \pi /3 \,\text {rad/s}. \end{aligned}$$

When the pendulum angle reached the inversion boundaries [$|\theta -\pi | < \pi/6$] rad with a small rotational speed ($|{\dot{\theta }}| < \pi$/3 rad/s), the inversion was considered as initiated ($inv_{begin}$), and the *score* started increasing proportionally (*k* = 19) to $|\theta -\pi |$. The inversion was considered to end once the pendulum fell outside of these conditions ($inv_{end}$). Within each experimental block, if the participant dropped the pendulum down, the score was retained until it was inverted again. At the beginning of a new block, the *score* and $\theta$ were reset to zero.

### Study protocol

The experiment was approved by the Cantonal Ethics Committee and the Swiss Agency for Therapeutic Products (Swissmedics) and followed the Declaration of Helsinki. We recruited 41 healthy right-handed participants for the study—evaluated with the Waterloo handedness questionnaire [41]—, but due to a data acquisition problem with one participant, only 40 participants were included in the data analysis (20 females, 20 males, age mean: 29, std.: 5.7 y.o.). All participants gave written consent to take part in the experiment.

Participants were randomly assigned to one of four training modalities—ten participants per modality, between 4 and 6 females per modality. Each training modality corresponded to combinations of two factors: Haptic Rendering (HR) and arm Weight Support (WS):

- Visual: Neither haptic rendering nor arm weight support was provided (HR:OFF, WS:OFF).
- Supported Visual: Arm weight support was provided, but not haptic rendering (HR:OFF, WS:ON).
- Visuo-Haptic: The haptic rendering of the pendulum dynamics was provided at the hand module, but not arm weight support (HR:ON, WS:OFF).
- Supported Visuo-Haptic: Arm weight support was provided in addition to haptic rendering (HR:ON, WS:ON).

The experiment consisted of two experimental sessions that were one to three days apart. Participants sat comfortably on a chair with a backrest and their right arms were attached to the exoskeleton cuffs with Velcro^®^ straps. The exoskeleton height and links lengths were adjusted for each participant. The experiment started with a short calibration phase ($\approx$ 2–3 min) to adjust the height of the virtual safety table just above the participant’s legs and to estimate the individual participants’ upper/lower arm weights.

**Fig. 3:**
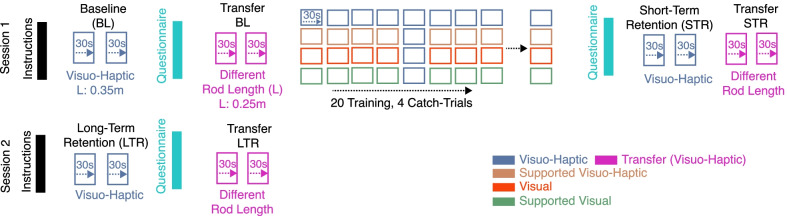
Study protocol. Participants were randomly assigned to one of four possible training modalities: Visuo-Haptic, Supported Visuo-Haptic, Visual, or Supported Visual. Training blocks (× 20) and catch-trials blocks (× 4) were used to assess the effect of providing haptic rendering and arm weight support on motor performance during training. Changes from baseline blocks (BL) to short-term retention (STR) and long-term retention blocks (LTR) were used to assess participants’ motor learning and had the Visuo-Haptic modality. Transfer learning was assessed with changes from transfer baseline blocks to short- and long-term transfer retention blocks, also with the Visuo-Haptic modality, but with a shorter pendulum (i.e., different dynamics). Participants’ sense of agency and motivation were assessed with questionnaires after baseline, training, and long-term retention blocks

The participants were instructed to swing the pendulum, to invert it and keep it inverted as long as possible. The instructions could be read on the screen. We included an exemplary video to facilitate the task understanding. After the instructions, participants performed two baseline blocks (BL) of 30 s each with the Visuo-Haptic modality (Fig. 3). Visuo-Haptic was chosen as the test modality as it was closest to reality—i.e., with haptics and no support. The participants then performed two transfer baseline blocks (TBL) (30 s each) with the Visuo-Haptic modality but with a *shorter pendulum*. The shorter pendulum length—0.25 m instead of 0.35 m—corresponded to a higher pendulum natural (swing) frequency, therefore, resulted in different pendulum dynamics. Participants rested their arms for 30 s between all experimental blocks.

After baseline, the training phase started. The training consisted of 24 experimental blocks of 30 s each. Twenty of these blocks were training blocks (T), in which participants trained with the modality according to the group they were assigned—either Visual, Supported Visual, Visuo-Haptic, or Supported Visuo-Haptic. The other four blocks were catch-trial blocks (CT) in which the participants trained with the Visuo-Haptic modality (as in baseline). The CT blocks were included to detect and remove potential learning effects from the training blocks during data analysis. The order of the CT blocks was uniformly distributed over time—5th, 9th, 13th, and 17th blocks—to counterbalance potential learning effects when comparing the training and catch-trial blocks. Participants rested their arms for 30 s between all training blocks.

Shortly after the last training block, participants performed a short-term retention test. Similar to the baseline test blocks, the participants performed two short-term retention blocks (STR) and two transfer short-term retention blocks with the Visuo-Haptic modality. The first experimental session lasted around 1 h.

The second experimental session consisted of only the long-term retention test. Participants performed two long-term retention blocks (LTR) and two transfer long-term retention blocks with the Visuo-Haptic modality.

The participants’ sense of agency and subjective motivation were assessed with questionnaires (see Additional file 1: Questionnaire for a complete list) after the first two baseline blocks, right after the last training block, and after the first two long-term retention blocks. To assess the sense of agency, we adapted three statements from the embodiment questionnaire from Piryankova et al. [42] to the pendulum task. Participants ranked their agreement with each of the three statements using a Likert scale between -3 (“strongly disagree”) and 3 (“strongly agree”). Twelve statements from the well-established Intrinsic Motivation Inventory (IMI, [43]) were used to assess the participants’ motivation. These statements were selected from the four subscales: interest/enjoyment, effort/importance, pressure/tension, and perceived competence. A Likert scale between 1 (“not at all”) and 7 (“very true”) was used for the answers. The questionnaire was presented in English. The (previous) responses given during the experiment were always visible for each participant to minimize the possibility that differences in participants’ memory skills confound the results [44].

### Outcome metrics

We used different metrics to analyze the participants’ task performance, movement, and physical effort. Each of these metrics provided one data point for one experimental block. Additionally, we used questionnaires to evaluate the participants’ level of agency and motivation.

#### Task performance

To evaluate how well participants performed the motor task—i.e., inverting the pendulum—the score was employed as the task performance metric. The score (eq. 3) increased depending on how vertically and still the pendulum was kept inverted, and for how long.

#### Movement strategy

To achieve a high score, one first needs to be able to lift the pendulum ball. This requires providing sufficient momentum to the pendulum ball. This can be achieved by making the pendulum pivot point lead the movement of the pendulum ball—i.e., when the pivot point applies a force to the ball through the rod.

The movement of the pivot point and the relative movement of the pendulum ball w.r.t. the pivot point are both almost cyclic. Thus, leading the pendulum ball corresponds to maintaining a positive phase difference between the pivot point movement and the relative movement of the pendulum ball w.r.t. the pivot point. To quantify this synchronicity, we first extracted the velocity of the pivot point and the relative velocity of the pendulum ball w.r.t. the pivot point in the horizontal direction, only during the time the pendulum was not yet inverted. We did not analyze the vertical direction because the analysis focuses on the part where the pendulum was not inverted, which ignores most of the data in the upward direction ($+z$), biasing the calculations. We then band-filtered the velocity time series to exclude the low-frequency drifts and high-frequency noise using second-order Butterworth bandpass filters with 0.5 Hz and 10 Hz cut-off frequencies. Next, the Hilbert transform was applied to the normalized velocity signals—with zero mean and unit standard deviation—and the phase signals (time-series) for the pivot point and the pendulum ball were obtained. Finally, the mean difference between the two phase signals within a block was calculated (movement phase difference). A positive movement phase difference is desired since it means the participant is leading the pendulum motion—i.e., the pivoting point is ahead of the pendulum ball. However, since the phase is defined only in the range between $-\pi$ and $\pi$, the optimum movement phase difference to achieve good performance is not known a priori, and needs to be checked with its correlation with task performance.

To evaluate how much participants explored the environment, we analyzed the participants’ movement variability with the standard deviation of the hand module position in both *y*—horizontal movement variability—and *z*—vertical movement variability—directions. Analyzing both directions independently allows for the evaluation of the potential direction-specific effects of weight support, which is acting only on the vertical direction, on the participants’ workspace variability.

#### Physical effort

The participants’ physical effort was estimated by norm averaging their joint torques. The generated joint torque was estimated by summing the recorded interaction torques and the torques needed by the participants to hold their arm at each position, online-calculated by the arm weight support algorithm [23].

#### Agency and motivation

The sense of agency and the four subscales of the IMI—i.e., interest/enjoyment, effort/importance, pressure/tension, perceived competence—had three questions each (Additional file 1: Questionnaire section). The averages of the three questions were calculated for each participant. Two participants did not answer one question each; therefore, the average of two questions was performed for the corresponding subscales.

### Statistical analysis

To evaluate potential differences during baseline between training groups (factor: Visual, Supported Visual, Visuo-Haptic, Supported Visuo-Haptic), we compared the baseline data—mean of the two baseline blocks—for each outcome metric between groups using one-way ANOVA.

To evaluate whether the task performance (score) is associated with the movement phase difference between the pivoting point and the pendulum ball, the relationship between the movement phase difference and the score was analyzed with Pearson correlations, separately at BL, STR, and LTR, with each participant providing one data point.

To check if the learning continued linearly during the whole training or whether it reached a plateau by the end of the training (e.g., logarithmic), we fit generalized linear models for the score metric with block number as a continuous time variable similar to [45], and tested whether the addition of the nonlinear part significantly improved the model.

To determine how providing Haptic Rendering (HR) and/or Arm Weight Support (WS) affected the participants’ performance during training (T), compared to not providing HR/WS, we first subtracted the learning effects—mean of catch trials (CT)—from the training performance—﻿mean of the 20 T blocks—for each outcome metric and participant, based on the assumption that learning curves were approximately linear. Then, the resulting T−CT values were analyzed with two-way ANOVAs (or two Kruskal-Wallis tests for the effects of HR and WS, respectively, if T−CT was non-normally distributed) with two factors: HR and WS; each factor having two levels: OFF and ON. If there was a significant (HR *x* WS) interaction, post hoc tests were performed to evaluate: the HR effect for WS:OFF and WS:ON; and the WS effect for HR:OFF and HR:ON.

To analyze the short-term learning effects of training with HR and/or WS, we took the difference of the short-term retention (STR) and baseline (BL) values—averaged between the two test blocks—of each outcome metric and participant. The effects of HR and WS on the STR−BL differences were analyzed either with two-way ANOVAs or with two Kruskal-Wallis tests, depending on the data distribution. For the analysis of the training modality on long-term learning, the same analyses were employed, but with the LTR−BL difference.

The short-/long-term effects of training with HR and/or WS on skill transfer were analyzed by comparing the differences between levels of HR and WS for the changes of score from baseline transfer to short-/long-term retention (STR/LTR) transfer using two-way ANOVAs. Furthermore, we analyzed if participants moved differently—i.e., with different movement variability and hand module speed—when performing the main task compared to the transfer task with a shorter pendulum rod at long-term retention. We used a mixed ANOVA for this comparison with pendulum length as a within-subject factor (levels: short, long), and training modality as the between-subjects factor.

The effect of HR and WS on participants’ agency and motivation levels after training (T−BL) and at long-term retention (LTR−BL) were analyzed using two-way ANOVA or two Kruskal-Wallis tests, depending on the questionnaire data distribution.

The normality of the data was visually inspected and evaluated using Kolmogorov-Smirnov tests from the Scipy module of Python. For one-/two-way ANOVAs, Afex package of *R* [46]; and for Kruskal-Wallis tests (used when the distributions were non-normal), Scipy module of Python [47] were employed. Bonferroni correction was used for multiple comparisons. The significance level was set to $\alpha$ = 0.05 for all statistical tests.

## Results

We did not find significant differences between training modalities during baseline for any of the outcome metrics.

The Pearson correlation between the absolute values of the movement phase difference and the score was not significant for baseline ($r= -0.181$, $p= 0.264$), but significant for short- ($r = 0.594$, $p < 0.001$) and long-term retention ($r = 0.336$, $p = 0.034$). This correlation suggest that the participants who achieved higher movement phase difference after training also achieved higher scores.

We found that the addition of nonlinear time variables to the generalized linear models—that represent logarithmic learning curves—did not improve the models significantly. This indicates that the learning was approximately linear and probably not completed after training ended. Furthermore, the maximum score that can be reached (by holding the pendulum vertically inverted for 30 s) is around 250, which no participants achieved so far.

### Effect of haptic rendering and weight support on participants’ performance during training

We did not find any significant effects of haptic rendering and weight support on the score during training (Additional file 1: Fig. S1, Results section).

**Fig. 4:**
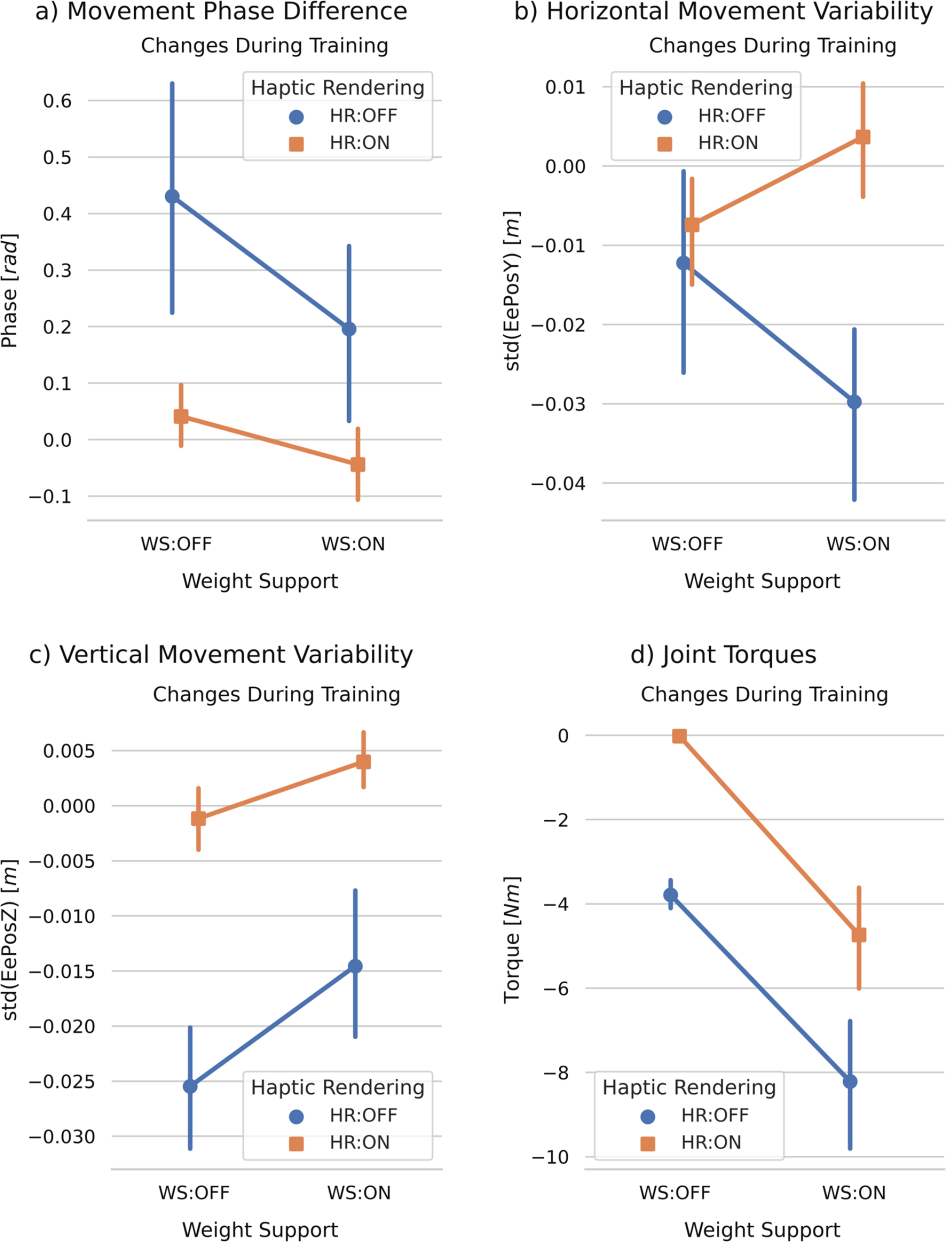
Effects of haptic rendering and weight support on participants’ outcome metrics during training. The differences between training blocks and catch-trial blocks, T−CT, are shown. The error bars indicate the 95% confidence interval

We found significant main effects of haptic rendering and weight support on the movement phase difference (Table 1). In particular, the addition of haptic rendering and/or weight support reduced the movement phase difference (Fig. 4a).

**Table 1:** Results (F and *p*-values) from the two-way ANOVAs (or Kruskal-Wallis tests if distributions were non-normal) from evaluating the effects of haptic rendering (HR) and arm weight support (WS) on the outcome metrics during training (T-CT), short-term learning (STR-BL), and long-term learning (LTR-BL)

	T−CT			STR−BL			LTR−BL		
	HR	WS	HR *x* WS	HR	WS	HR *x* WS	HR	WS	HR *x* WS
Score	(3.36) 0.075	(1.39) 0.245	(0.38) 0.539	**(6.13) ** **0.018 **	(2.71) 0.108	(0.24) 0.628	**(8.47)** **0.006**	**(6.05)** **0.019**	(1.21) 0.280
Movement phase difference	**(18.32)** <**0.001**	**(4.74)** **0.036**	(1.03) 0.316	**(9.76)** **0.004**	**(4.62)** **0.038**	(0.01) 0.939	**(8.36)** **0.006**	**(5.43)** **0.026**	(1.09) 0.303
Horizontal movement variability	**(12.35)** **0.001**	(0.35) 0.556	**(6.90)** **0.013**	(0.71) 0.403	(0.20) 0.654	(0.82) 0.372	(0.16) 0.688	(1.81) 0.187	(0.76) 0.389
Vertical movement variability	**(74.90)** <**0.001**	**(10.52)** **0.003**	(1.36) 0.251	(1.29) 0.263	(0.02) 0.897	(0.15) 0.704	(0.19) 0.666	(0.31) 0.583	0.22 0.641
Joint torques	**(44.74)** <**0.001**	**(71.28)** <**0.001**	(0.07) 0.789	(0.59) 0.449	(0.42) 0.523	(<0.01) 0.955	(H(1)=0.70) 0.402	(H(1)=0.39) 0.534	–

	T−BL			STR−BL			LTR−BL		
	HR	WS	HR *x* WS	HR	WS	HR *x* WS	HR	WS	HR *x* WS
Transfer task score	–	–	–	**(8.72)** **0.006**	(1.60) 0.214	(2.51) 0.121	**(4.64)** **0.038**	(2.11) 0.155	(0.85) 0.362
Sense of agency	(0.11) 0.741	(1.56) 0.220	(0.56) 0.459	–	–	–	(0.86) 0.360	(0.69) 0.412	(0.19) 0.663
Interest/enjoyment	(0.19) 0.662	(1.38) 0.247	(0.54) 0.467	–	–	–	(0.03) 0.857	(1.06) 0.310	(1.62) 0.212
Effort/importance	(0) 1.000	(2.14) 0.153	(1.37) 0.218	–	–	–	(0.75) 0.392	(1.08) 0.305	(0.03) 0.863
Pressure/tension	(0.54) 0.468	(0.02) 0.884	(3.10) 0.087	–	–	–	(0.54) 0.468	(0.54) 0.468	(0.08) 0.779
Perceived competence	(1.41) 0.243	(2.37) 0.133	(0.33) 0.571	–	–	–	(1.35) 0.254	(3.96) 0.054	(0.44) 0.511

F values—or H values if Kruskal-Wallis tests were used—are indicated in brackets for F(0,36)—or H(1). Significant *p*-values are indicated in bold font

We found a significant interaction between haptic rendering and weight support in the horizontal movement variability during training (Table 1). In particular, when participants practiced with weight support, the addition of haptic rendering increased the horizontal movement variability (t(36) = 4.34, $p<$ 0.001, Fig. 4b). Both practicing with haptic rendering and weight support resulted in significantly greater vertical movement variability when compared to practicing without them (Fig. 4c, Table 1).

Finally, we found significant main effects for both haptic rendering and arm weight support on the joint torques (Table 1). While the addition of haptic rendering increased the forces participants’ created, the addition of weight support reduced them (Fig. 4d).

### Effects of haptic rendering and weight support on motor learning and skill transfer

**Fig. 5:**
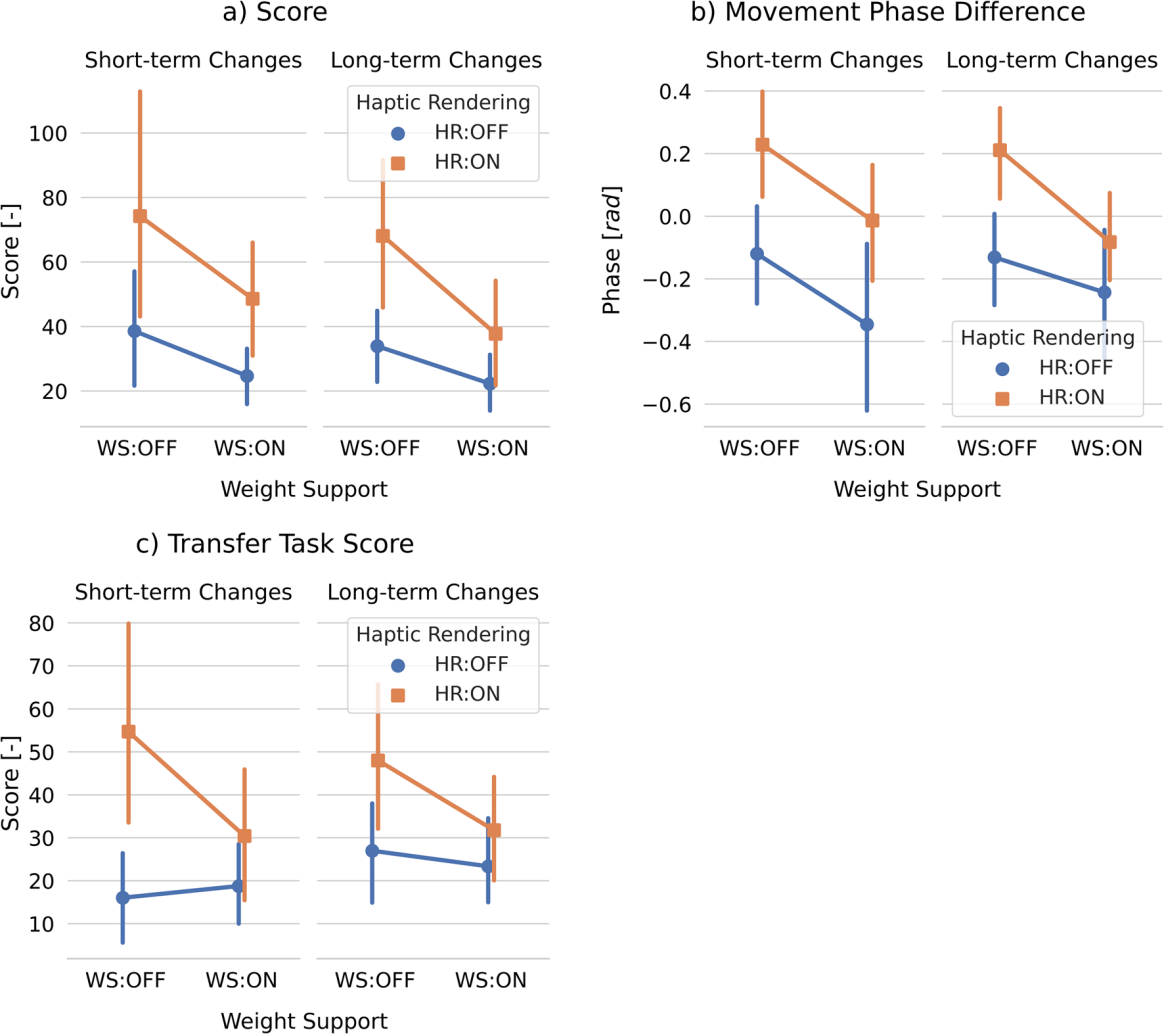
Effects of haptic rendering and weight support on participants’ motor learning and skill transfer. Short-/Long-term changes correspond to the differences between short-/long-term retention blocks and baseline blocks: (STR−BL) and (LTR−BL). The error bars indicate the 95% confidence interval

We found a significant main effect of haptic rendering on the score change from baseline to short-term retention and long-term retention (Table 1, Fig. 5a). We also found a significant main effect of weight support on long-term learning. While the addition of haptic rendering enhanced short- and long-term learning, the addition of weight support hampered learning.

We found significant main effects of haptic rendering and weight support on the movement phase difference changes from baseline to short- and long-term retention (Table 1). While the addition of haptic rendering increased the movement phase difference, the addition of weight support decreased it (Fig. 4b).

Training with haptic rendering also significantly enhanced short- and long-term skill transfer—i.e., increased the transfer task score—compared to training without haptics (Table 1; Fig. 5c). Furthermore, we found that participants made less variable (in the horizontal direction; F(1,36) = 9.34, *p* = 0.004) and faster hand movements (F(1,36) = 7.63, *p* = 0.009) with the short pendulum rod (transfer task) compared to the long pendulum rod (main task) during long-term transfer.

### Effects of haptic rendering and weight support on agency and motivation

We did not find significant effects of haptic rendering and weight support on participants’ sense of agency nor in any of the IMI subscales for the changes from baseline to after training, nor at long-term retention (Table 1, Additional file 1: Fig. S2, Results section).

Overall, the mean sense of agency was high during training (1.7 over 3) and at long-term retention (2.0). The mean interest/enjoyment and effort/importance were also high (5.6 and 5.5 over a maximum of 7) throughout the experiment (Additional file 1: Fig. S2, Results section).

## Discussion

We found that training with haptic rendering was more effective, compared to training without haptics, for learning a dynamic task and transferring the acquired skills to a different dynamic system. Training with assistance, on the other hand, hampered learning. In discussing these results, we return to the seven hypotheses stated in the “Introduction” that we aimed to test with our experiment.

### Haptic rendering encourages workspace exploration and increases physical effort during training

We hypothesized that haptic rendering would encourage participants’ workspace exploration—i.e., higher movement variability—and increase their physical effort during training compared to training without haptics. The addition of somatosensory information regarding the interaction forces with virtual tangible objects through haptic rendering has been suggested to change both the information and power exchange between the participants and the virtual training environment [48]. Such changes might alter participants’ movement strategies during task performance as a result of new aspects to be learned—e.g., predicting the task dynamics—and/or changes in the nature of the robot-human interaction—i.e., the inclusion of interaction forces.

We confirmed that when haptic rendering is added during training, the participants’ vertical movement variability significantly increases compared to training without haptics. The higher movement variability can be interpreted as an increase in workspace exploration as participants cover more workspace. One explanation might be that haptic rendering prompts participants to not only explore the kinematic behavior of the pendulum but also to predict and compensate for the interaction forces from its dynamics [49], which might require more exploration to learn.

We also confirmed that adding haptic rendering significantly increases the participants’ physical effort, estimated as joint torques. The increase in the participants’ physical effort is probably due to the forces from the pendulum dynamics projected on the participants’ hands—e.g., the pendulum weight and centrifugal force—that the participants need to compensate to achieve the task.

Nevertheless, while haptic rendering affects the participants’ movement strategy, it does not improve their task performance (score) during training. Probably, exploiting the haptic information to enhance the task performance is a slow process that requires learning, unlike the short-term but temporary benefits on the training performance observed in other haptic assisting methods [29].

### Weight support decreases physical effort during training and encourages workspace exploration

We expected that arm weight support would reduce participants’ physical effort during training compared to non-assistance and would promote workspace exploration since participants do not have to bear the weight of their own arms.

As expected, training with arm weight support reduces the participants’ physical effort significantly. This is in line with previous research that found reduced levels of muscle activity during movement in healthy young participants [50], elderly participants [35], and stroke patients [34].

We also found that weight support significantly enhances workspace exploration, but only in the vertical movement direction. The increase in the vertical movement variability could be explained by the facilitated vertical arm movements resulting from the reduction of participants’ effort to counterbalance their arms against gravity. However, contrary to previous literature on stroke patients [37], we did not find a significant main effect of weight support on the horizontal movement variability during training. We see this as an opportunity, rather than a limitation, since in most normal everyday tasks, the arm movements are predominantly in the vertical plane [51]. Thus, as hypothesized by Krakauer and Carmichael in [52], the provision of weight support in 3D seems to allow the exploration and practice of movements that are functional and useful to many activities of daily living.

### Arm weight support together with haptic rendering enhances horizontal workspace exploration

An important problem with robotic assistance methods is that the assisting forces might alter the perceived task dynamics provided through haptic rendering via the same actuators [30]. Therefore, robotic assistance, if not applied adequately, could hamper any potential benefits of haptic rendering on participants’ movements. We proposed that using robotic assisting methods that do not constrain the participants’ movement might be a good strategy to prevent the interference between haptic rendering and assistance. Arm weight support does not depend on the movement [40] and does not enforce a fixed trajectory [24]. It has been shown to reduce the average muscle activation level without affecting the general patterns of muscle activation in healthy young individuals [50], healthy elderly individuals [35], and stroke patients [34]. Moreover, there is evidence that the nervous system processes static (e.g., gravitational) and dynamic (e.g., inertial) forces separately [53, 54], which hints that the compensation of the pendulum haptic rendering forces could be separated from the arm weight support, which is purely static [55]. Arm weight support only counterbalances the arm weight with a fixed magnitude through the workspace, and therefore, perceiving the dynamic haptic rendering would still be possible, especially in the directions perpendicular to gravity. Therefore, we did not expect any significant interaction between the haptic rendering and arm weight support on the participants’ movements.

Contrary to our expectations, we found a significant interaction between the arm weight support and haptic rendering on the horizontal movement variability during training. One reason for this interaction could be that the static part of the pendulum haptic rendering forces—i.e., gravitational forces—was high enough to interact with the arm weight support, which is also static by design. This interaction might be, however, beneficial, as the addition of weight support on top of the haptic rendering enhances the workspace exploration in the horizontal direction. This could be due to the weight support increasing the participants’ force-generating capacity, as the weight support reduces the participants’ effort during training. Thus, weight support seems to promote haptic rendering-induced workspace exploration, which might be beneficial for motor learning [24, 25].

### Training with haptic rendering enhances motor learning

We hypothesized that training with haptic rendering would enhance motor learning and skill transfer due to enhanced workspace exploration. Furthermore, feeling the haptic forces from the pendulum would provide extra multisensory information about the highly dynamic task compared to training without haptics, resulting in participants learning to better synchronize their movements with the pendulum dynamics, and ultimately, enhancing learning. The results support our hypothesis: The provision of haptic rendering during training enhances motor learning in both short- and long-term retention.

We argue that several rationales might be behind the positive influence of haptic rendering on motor learning. First, predicting the interaction forces with a virtual object requires learning an internal model of the object dynamics [56]. Haptic rendering might facilitate the acquisition of this internal model during training by increasing the workspace exploration, e.g., by promoting workspace variability and prompting participants to discover better movement strategies to invert the pendulum. The positive effect of workspace exploration on motor learning has already been reported in previous studies that showed an association between higher movement variability and accelerated motor learning [24–26].

Secondly, the addition of haptic information on top of the visual information might provide more enriched multimodal information about the dynamic environment, compared to only visual feedback, which may enhance the learning of this especially complex task [18, 19, 57]. The haptic rendering provides extra information about the weight of the pendulum through the magnitude of the rod force, and about the pendulum angle through the direction of the force. The amount of task-relevant information conveyed by the haptic rendering on top of the visual feedback might, therefore, reduce the conditional task difficulty—i.e., the challenge presented to the learner—and, in line with the challenge point framework [58], enhance motor learning.

The enhanced motor learning observed after training with haptic rendering might also be related to the acquisition of a better motor strategy to invert the pendulum. We found that the absolute values of the score and movement phase difference between the pendulum ball and the hand module are positively correlated during short- and long-term retention. This correlation suggests that high movement phase difference is associated with high task performance. Importantly, we found that training with haptic rendering significantly increases the participants’ ability to synchronize their movement with the movement of the pendulum ball in order to maintain a high movement phase difference. As the pull force that the participants feel when haptic rendering is applied depends on their movement phase difference through the inertial forces, participants probably were more aware of their movement phase difference than participants who trained without haptic rendering. This extra information might have helped them to discover and maintain more efficient movement phase differences, which resulted in high scores after training.

### Training with haptic rendering enhances skill transfer

As hypothesized, participants who trained with haptic rendering generalized the acquired skill to the transfer task to a greater extent than participants who trained without haptics. Only a few studies have evaluated motor skill transfer from training in a virtual environment with only visual feedback to more realistic environments in healthy and neurologically impaired populations (see [7] for a review). The majority of the studies reviewed by Levac et al. found that the internal models acquired during training in virtual environments do not generalize to more realistic tasks that also incorporate haptics (e.g., [49, 59]).

In their review [7], Levac et al. discussed that the lack of skill transfer is, among others, due to the lack of haptic input in the virtual task. This lack of haptic rendering prompted participants to rely only on the provided visual information during training, resulting in different perceptual-motor couplings compared to more realistic tasks. According to the specificity of learning hypothesis [60], the optimal source of sensory information is used to perform a movement and it is expected that skill transfer is enhanced when the training conditions are similar to the conditions of real-life performance [61]. In our experiment, training with haptic rendering allowed participants to experience and learn to compensate for the interaction forces from the haptic rendering and they probably employed the acquired skill to handle external forces to successfully perform the transfer task.

Levac et al. also attributed the limited skill transfer to the differences in participants’ movement kinematics performed in the virtual vs. real environments with complex dynamics. While we did not test for a “real” version of the pendulum task (without a robot), our transfer task with a shorter pendulum could be considered different enough from the main trained task with haptics. Differences in the dynamical properties of virtual objects (i.e., pendulum length) were found to affect the movement strategy adopted by individuals when manipulating dynamic objects [62, 63]. In our experiment, the transfer task consisted of inverting a pendulum with a shorter rod length than the one used in the test task. We found that participants move differently to achieve the transfer task, i.e., with less variable and faster movements, compared to the main task. This difference in kinematics was expected based on previous literature [62, 63], as pendulums with longer rods are inherently more stable than short-rod pendulums. Nevertheless, although participants in the haptic rendering group showed different movement kinematics between the virtual and transfer tasks, the similar sensorimotor integration experienced during training with haptic information [7, 17] probably facilitated skill transfer.

### Weight support hampers motor learning

We hypothesized that arm weight support would enhance motor learning due to a more efficient allocation of participants’ effort into the dynamic environment and reduction of participants’ fatigue. However, contrary to our expectations, training with arm weight support significantly hampers long-term motor learning compared to training without assistance. Importantly, weight support significantly decreases the participants’ ability to synchronize their movement with the pendulum ball—i.e., reduces the movement phase difference—in short- and long-term retention, which we found to be correlated with good task performance (high score).

Our findings are inconsistent with previous studies that showed enhanced motor learning when arm weight support is provided during training, e.g., in children with cerebral palsy [64] and stroke patients [65]. However, to our knowledge, no studies have examined the effect of arm weight support in learning complex motor tasks in healthy participants. An interpretation for these unforeseen results is that the weight support disrupts motor learning because participants rely on the assistance during training and fail to learn the motor commands required to perform the desired task unassisted at retention [66]. The weight support level provided to participants—100 $\%$ of their arm weight—might have been excessive. Perhaps, lower levels of weight support and even weight support levels that adapt according to the ongoing performance of the participants [67, 68], might have yielded better learning results.

Furthermore, the arm weight support constantly pushes the arm up to counterbalance gravity. Thus, healthy participants might overshoot during training when they move in the vertical direction as they have an internal model of their own arm dynamics that does not include the support of the robot. This idea is supported by the observed increase of the vertical movement variability during training with weight support. The highly dynamic and unstable nature of the pendulum task and the fact that the complex dynamic task requires to work against gravity probably exacerbated this problem.

We also expected that weight support would enhance motor learning due to a reduction of participants’ fatigue. Although we found that participants’ effort decreased when weight support was added during training, little can be said about their level of fatigue. The quantification of the physical effort through the norm of joint torques was only an estimation and we did not measure the fatigue directly, e.g., with electromyography [69]. As participants were allowed to rest between experimental task blocks (30 s rest between each block), this might have been sufficient to prevent participants’ fatigue, even in the absence of weight support.

### Training with haptic rendering and weight support does not affect agency and motivation

Finally, we expected that the inclusion of haptic rendering during training would enhance the participants’ level of agency and motivation. The sense of agency—i.e., the subjective feeling of being in control over own actions [70]—is associated with the ability to accurately predict the consequences of the own actions [71] and the movements of the objects one interacts with [72]. We expected that haptic rendering would support the formation of the internal model of the pendulum through reinforced sensorimotor integration, allowing for a more accurate prediction of the pendulum motion, ultimately enhancing participants’ agency.

However, we did not observe any significant effects of haptic rendering or weight support on the participants’ agency, neither during training nor in long-term retention. The level of agency reached high values regardless of the training modality already during training. Therefore, ceiling effects may have prevented us from detecting significant effects in the long-term retention. Questionnaires assessing agency in the middle of the training phase, rather than at the end, might have been able to show/capture differences between training modalities.

Furthermore, the addition of haptic information has been shown to enhance the level of immersion—closely related to the subjective feeling of presence—in virtual environments [73]. Higher immersion has been reported to enhance motivation by lowering the pressure/tension during training in virtual reality [22]. Therefore, we hypothesized that haptic rendering would enhance participants’ motivation through higher immersion.

However, contrary to our expectations, training with haptic rendering did not affect the motivation level significantly—assessed on four subscales: pressure/tension, interest/enjoyment, effort/importance, and perceived competence—compared to training without haptics. Similar to the perceived agency, participants’ mean interest/enjoyment and effort/importance were high during the entire experiment, resulting in a potential ceiling effect [74]. Furthermore, the inter-personal variances in the pressure/tension and perceived competence metrics probably masked the potential differences across modalities. Nevertheless, we observed a trend for a smaller increase of the perceived competence in long-term retention after training with arm weight support, compared to training without assistance. The smaller performance gains from baseline to long-term after training with arm weight support compared to training without assistance likely prevented participants from experiencing higher levels of competence.

### Lessons learned and implications for functional robot neurorehabilitation

Although our study was performed with healthy participants, motor recovery is generally accepted to be a form of motor (re)learning [75]. Therefore, our findings might have important implications for the neurorehabilitation of brain-injured patients.

One of the main aims of neurorehabilitation is to enhance patients’ capabilities to successfully perform ADLs independently [7]. Therefore, it is important that the improvements observed in motor performance after robot-assisted training transfer to functional movements. A significant number of ADLs involve the physical interaction with environments with complex dynamics, such as carrying a cup of coffee or watering plants [8]. Our findings indicate that the provision of haptic rendering during robot-assisted training enhances healthy individuals’ learning of motor tasks that require manipulating objects with complex dynamics and promotes skill transfer to tasks with different dynamics. Therefore, including haptic rendering during robotic neurorehabilitation might indeed provide a multisensory enriched environment for training, and consequently promote functional gains [28]. However, patients with cognitive and sensory impairments might find challenging the perception and interpretation of the haptic rendering forces [76]. Future research is needed to investigate how haptic rendering should be designed to support the neurorehabilitation of brain-injured patients [28].

Our results also indicate that arm weight support hampers motor learning and skill transfer for this complex task in healthy young participants. While not providing robotic assistance during training might be possible in healthy individuals, stroke patients might need physical support to train sensorimotor skills with high intensity [4]. Therefore, providing arm weight support might still enhance neurorehabilitation in patients by enabling them to practise movements. Moreover, intermediate and/or adaptive levels of arm weight support might provide good trade-offs between enabling patients to train complex motor skills and limiting the detrimental effects of excessive physical robotic assistance on motor learning. In future studies, we will investigate whether adaptive weight support might be beneficial for learning complex motor skills.

### Study limitations

The first limitation of our study is that the test blocks—baseline, short- and long-term retention—were only performed with the Visuo-Haptic modality. We did not include test blocks with different modalities—e.g., Visual—because we aim to evaluate the effect of training with haptic rendering and arm weight support on a task that is close to reality, namely in the presence of haptics. However, we did not test whether the observed effects of haptic rendering and arm weight support on motor learning would generalize to practicing the test and transfer tasks without haptics. We decided to keep the study relatively short to prevent participants to get unmotivated and exhausted and to avoid that participants already learned the task during baseline.

A second study limitation is that we did not have direct measurements of the participants’ physical effort and fatigue. Electromyography [69] and periodic measurements of maximum voluntary contraction (measurement of muscle strength [77]) are common methods to measure participants’ effort and fatigue. However, the employment of these techniques would have increased the experiment duration, which might negatively affect participants’ motivation, and possibly motor learning. Therefore, we estimated the participants’ physical effort through the average norm of joint torques, similarly to [78].

A final study limitation is that we did not perform the study with brain-injured patients but with health young participants. This limits the extent to which our study findings can be generalized to brain-injured patients. Patients might not be able to perform the required movements to fulfill the task without physical support, and thus, arm weight support could have, indeed, positive effects on motor learning, in contrast to our study findings obtained in healthy young participants.

## Conclusion

We demonstrated that training with haptic rendering of virtual environments enhances motor learning and skill transfer of dynamic tasks compared to training without haptic rendering. The additional somatosensory information provided through haptic rendering increases workspace exploration and physical effort during training, allows participants to get better at synchronizing their movements with the dynamics of the object they interact with, and may enforce the resemblance to real-life training. Consequently, the enhanced learning associated with haptic rendering is generalized to different dynamic systems. If employed in robotic neurorehabilitation, haptic rendering might promote functional gains and enhance the transfer of clinical improvements into activities of daily living.

We also demonstrated that the assisting forces from human arm weight support hamper motor learning in young healthy participants. Although weight support increases participants’ workspace exploration during training, it also prevents them from coordinating their movements with the object dynamics. Future research should investigate intermediate and adaptive levels of robotic assistance to prevent the adverse effects of excessive physical assistance on motor learning of health participants and neurorehabilitation of brain-injured patients.

## Supplementary Information

**Additional file 1: ** Questionnaire and results.

## Data Availability

The dataset supporting the conclusions of this article is available in the [Zenodo] repository, https://doi.org/10.5281/zenodo.5109743.
