# Supplementary material for: Towards functional robotic training: motor learning of dynamic tasks is enhanced by haptic rendering but hampered by arm weight support
Source: J Neuroeng Rehabil. 2022 Feb 13;19:19. doi: 10.1186/s12984-022-00993-w (PMC8842890; doi:10.1186/s12984-022-00993-w)
Supplement: Supplementary file 1 — Additional file 1: Questionnaire and results. [file 12984_2022_993_MOESM1_ESM.pdf]

RESEARCH

Additional File 1

Özhan Özen[1*], Karin A Buetler[1] and Laura Marchal-Crespo[1,2]

*Correspondence: ozhanozen@hotmail.com
[1]Motor Learning and Neurorehabilitation Laboratory, ARTORG Center for Biomedical Engineering Research, University of Bern, Freiburgstrasse 3, 3010 Bern, Switzerland
Full list of author information is available at the end of the article

Questionnaire

Agency:
- It seemed like I was in control of the pendulum.
- It seemed as if the pendulum was controlling me.
- It seemed like I was causing the movements of the pendulum.

Interest/Enjoyment:
- I thought this activity was quite enjoyable.
- The task was fun to do.
- I would describe this activity as very interesting.

Effort/Importance:
- I tried very hard on this activity.
- I put a lot of effort into this.
- It was important to me to do well at this task.

Pressure/Tension:
- I felt pressured while doing these.
- I was anxious while working on this task.
- I felt very tense while doing this activity.

Perceived Competence:
- I was pretty skilled at this activity.
- I am satisfied with my performance at this task.
- I think I am pretty good at this activity.

Results

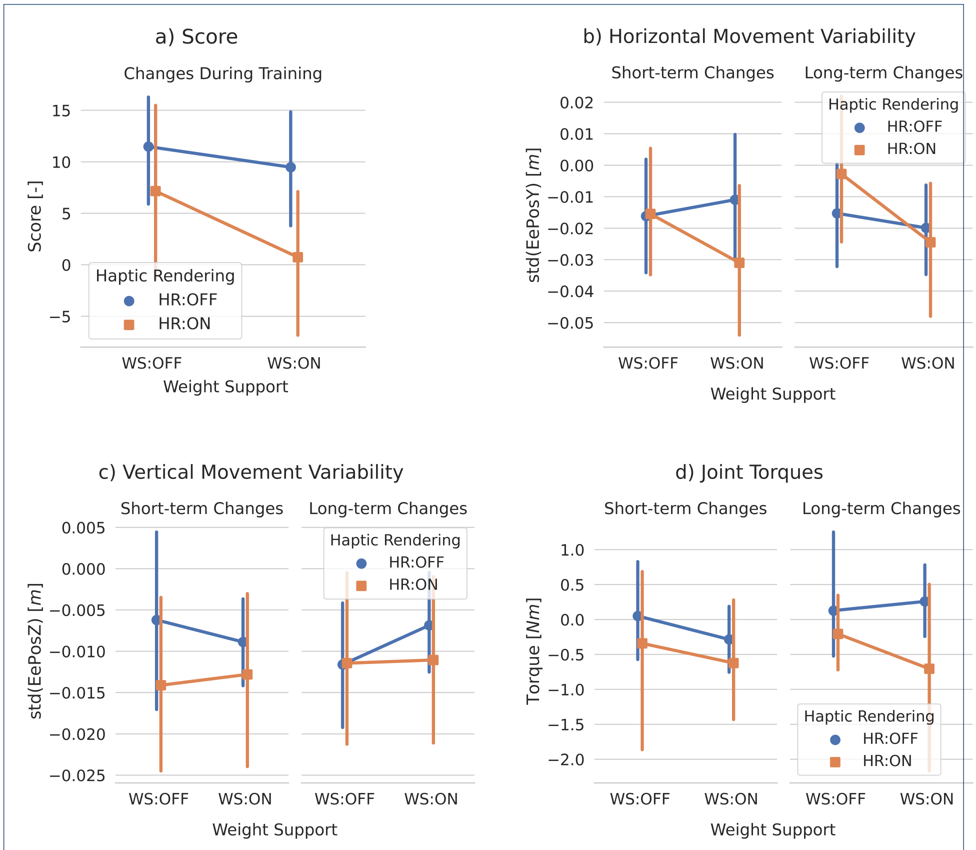

Figure S1 Effects of haptic rendering and weight support on participants' outcome metrics during training, and at short-/long-term retention. Changes during training correspond to the differences between training blocks and catch-trial blocks, T−CT. Short-/Long-term changes correspond to the differences between short-/long-term retention blocks and baseline blocks: (STR−BL) and (LTR−BL). a) Score. b) Horizontal movement variability. c) Vertical movement variability. d) Joint torques. The error bars indicate the 95% confidence interval.

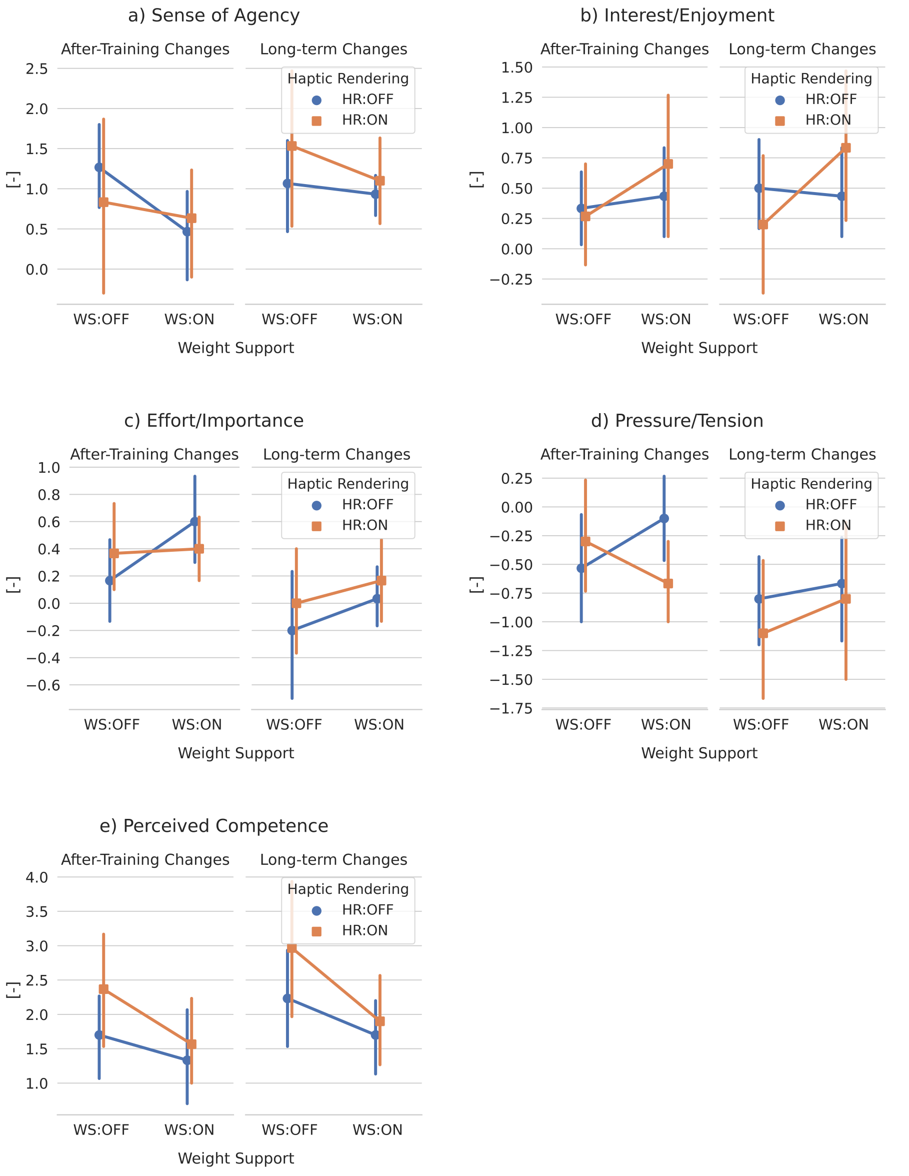

Figure S2 Effects of haptic rendering and weight support on participants' self-reported values of sense of agency and intrinsic motivation after training and long-term retention. After-training/Long-term changes correspond to the differences between training/long-term retention blocks and baseline blocks: (T−BL) and (LTR−BL). (a) Interest/Enjoyment. (b) Perceived competence. (c) Effort/Importance. (d) Pressure/Tension. Likert scales are based on [1] for agency and [2] for intrinsic motivation metrics. The error bars indicate the 95% confidence interval.

Author details
[1]Motor Learning and Neurorehabilitation Laboratory, ARTORG Center for Biomedical Engineering Research, University of Bern, Freiburgstrasse 3, 3010 Bern, Switzerland. [2]Department of Cognitive Robotics, Delft University of Technology, Mekelweg 2, 2628 CD Delft, the Netherlands.

References

1. Piryankova, I.V., Wong, H.Y., Linkenauger, S.A., Stinson, C., Longo, M.R., Bülthoff, H.H., Mohler, B.J.: Owning an overweight or underweight body: Distinguishing the physical, experienced and virtual body. PLoS ONE **9**(8), 103428 (2014). doi:10.1371/journal.pone.0103428
2. Ryan, R.M., Connell, J.P., Plant, R.W.: Emotions in nondirected text learning. Learn. Individ. Differ. **2**(1), 1–17 (1990). doi:10.1016/1041-6080(90)90014-8
